# CD2068 potentially mediates multidrug efflux in *Clostridium difficile*

**DOI:** 10.1038/s41598-017-10155-x

**Published:** 2017-08-30

**Authors:** Chawalit Ngernsombat, Suthasinee Sreesai, Phurt Harnvoravongchai, Surang Chankhamhaengdecha, Tavan Janvilisri

**Affiliations:** 1 Department of Biochemistry, Faculty of Science, Mahidol University, Bangkok, 10400 Thailand; 2 Department of Biology, Faculty of Science, Mahidol University, Bangkok, 10400 Thailand

## Abstract

*Clostridium difficile* is a major cause of antibiotic-associated diarrhea and the treatment thereof becomes more difficult owing to a rise of multidrug resistant strains. ATP-binding cassette (ABC) transporters are known to play a crucial role in the resistance to multiple antibiotics. In this study, the potential contribution of an ABC transporter in *C. difficile* multidrug resistance was investigated. The expression level of the *cd2068* gene in *C. difficile* encoding an ABC transporter was up-regulated following the exposure to certain antibiotics compared to the control cells. Heterologous expression of CD2068 in *Escherichia coli* revealed that it mediated the efflux of fluorescent substrates and conferred resistance to multiple drugs. The CD2068-associated ATPase activity in membrane vesicles was also stimulated by various antibiotics. Furthermore, the insertional inactivation of the *cd2068* gene in *C. difficile* led to a significant increase in susceptibility to antibiotics, which could be genetically complemented, supporting that CD2068 was directly associated to the drug resistance. These results demonstrate the potential role for the ABC transporter CD2068 in the resistance mechanism against multiple drugs in *C. difficile*.

## Introduction

*Clostridium difficile* is one of the prominent causes of infectious nosocomial diarrhea and is responsible for ~25% of human antibiotic-associated diarrhea^1^. Although patients with *C. difficile* infection (CDI) may acquire the organism from the environment^2^, potential sources of CDI in humans may include domestic and farm animals since an overlap between isolates from humans and animals has been demonstrated^3, 4^. CDI causes diseases ranging from mild diarrhea to life-threatening pseudomembranous colitis. The number of incidence and the level of severity of CDI has markedly increased across the continents^5, 6^. Antimicrobial treatment plays an important role in the progression of CDI. It has been postulated that the antibiotic therapy disrupts normal gut microbiota, allowing *C. difficile* colonization and growth because it is naturally resistant to many drugs used to treat other infections, thereby enabling its toxin production^7^. The first-line therapy for CDI includes metronidazole and vancomycin, however, the resistance to these drugs has been reported, rendering the treatment ineffective^8^. In recent years, the antimicrobial therapy for CDI tends to be more difficult due to the development of multidrug resistant (MDR) strains including the hypervirulent drug resistant strain, BI/NAP1/027^9^. A number of highly virulent MDR strains belonging to other ribotypes have also been described^10, 11^.

Multidrug resistance in *C. difficile* continues to plague antimicrobial chemotherapy of CDI, posing a major cause of concerns within healthcare and hospital environments^12^. The clinical impact of resistance is immense, characterized by increased cost, length of hospital stays and mortality. Certain mechanisms to avoid antimicrobial therapy in *C. difficile* have been reported including target modification (e.g. GyrA/GyrB mutations)^13^, inactivation of drugs (e.g. erythromycin ribosomal methylases)^14^, enzymatic degradation (e.g. ß- lactamases)^15^. However, these mechanisms are only specific to one class of drugs. The main strategy to protect the cells from multiple drugs is believed to be associated with the active efflux on the plasma membrane mediated by ATP-binding cassette (ABC) transporters^16, 17^. ABC transporters belong to a ubiquitous protein superfamily in all domains of life, which transport a variety of substances ranging from simple ions to large molecular weight molecules. Generally, an ABC transporter consists of two nucleotide binding domains (NBD) and two transmembrane domains (TMD). Translocation of the substrates initiates when the transporter hydrolyzes ATP at the NBD^17^ with an exception of recent finding on the assistance of proton coupling^18^. The energy is required to induce conformational alternation of the TMD, thereby allowing a drug to translocate across the cell.

ABC transporters have been closely linked to multidrug resistance in various microorganisms, such as the multidrug transporter LmrA in a gram-positive bacterium, *Lactococcus lactis* ^19^, and ATP binding protein VgaB and MsrA in *Staphylococcus aureus* ^20, 21^. Among the genus of Clostridium, an ABC transporter, CmpA, in *C. hathewayi* has previously been shown to be involved in fluoroquinolone resistance^22^. The characterization of another ABC transporter, CPE1506, in *C. perfringen*s revealed a high level of homology to CmpA, supporting the contribution of ABC transporter on MDR^23^. A predicted ABC transporter operon has also been reported in *C. difficile*, which has been associated with the resistance to cationic antimicrobial peptides^24^. To date, the role and mechanism of ABC transporters in multidrug resistance in *C. difficile* have not been established. In the present investigation, we identified and characterised an ABC transporter that appears to play an important role in mediating the efflux of multiple drugs in *C. difficile*.

## Results

### Antibiotics induced *cd2068* gene expression in *C. difficile*

**Figure 1:**
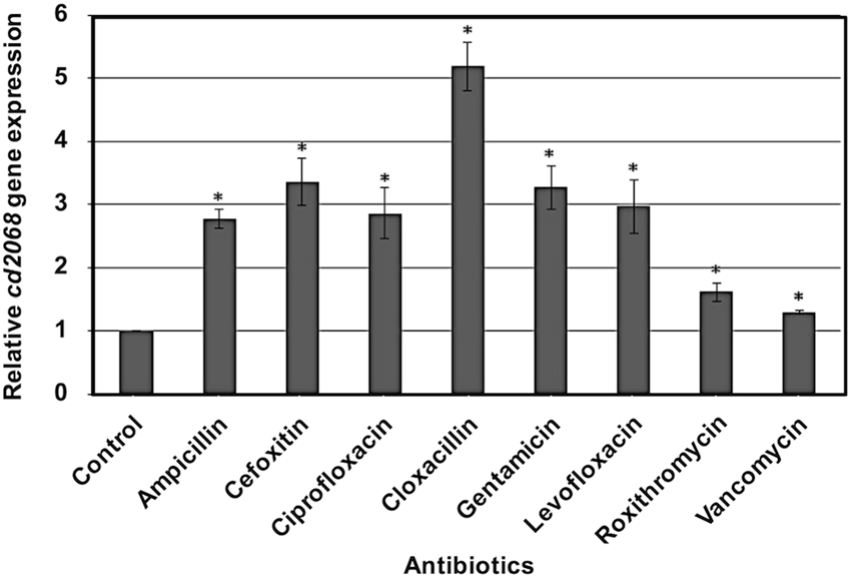
Antibiotics induced *cd2068* gene expression in *C. difficile. C. difficile* 630 were grown in the presence of various antibiotics; 1 µg/ml ampicillin, 25 µg/ml cefoxitin, 1 µg/ml ciprofloxacin, 25 µg/ml cloxacillin, 10 µg/ml gentamicin, 1 µg/ml levofloxacin, 60 µg/ml roxithromycin, and 0.25 µg/ml vancomycin. The relative *cd2068* gene expression was calculated as described in Methods. The error bars and the asterisks represent the standard errors and the statistical significance with the *p* values < 0.05, respectively.

Transcriptomic data of *C. difficile* revealed the up-regulation of certain genes encoding putative ABC transporters upon the exposure to tested drugs^25^. Among them, *cd2068* was selected for further investigation on its potential role in antibiotic resistance. Quantitative PCR was carried out to further determine the level of the *cd2068* gene expression following exposure to a range of antibiotics. The results revealed that the expression of *cd2068* was induced in the antibiotic-treated cells compared to that observed in the control cells without antibiotic treatment (Fig. 1). The highest level of induction was observed when the cells were exposed to cloxacillin (~5.2 fold). The increases in gene expression were comparable when the cells were exposed to ampicillin, cefoxitin, ciprofloxacin, gentamicin, and levofloxacin. Roxithromycin and vancomycin were also significantly able to induce the *cd2068* gene expression with the level of less than 2-fold compared to the controls.

### CD2068 conferred drug resistance in *Escherichia coli*

**Figure 2:**
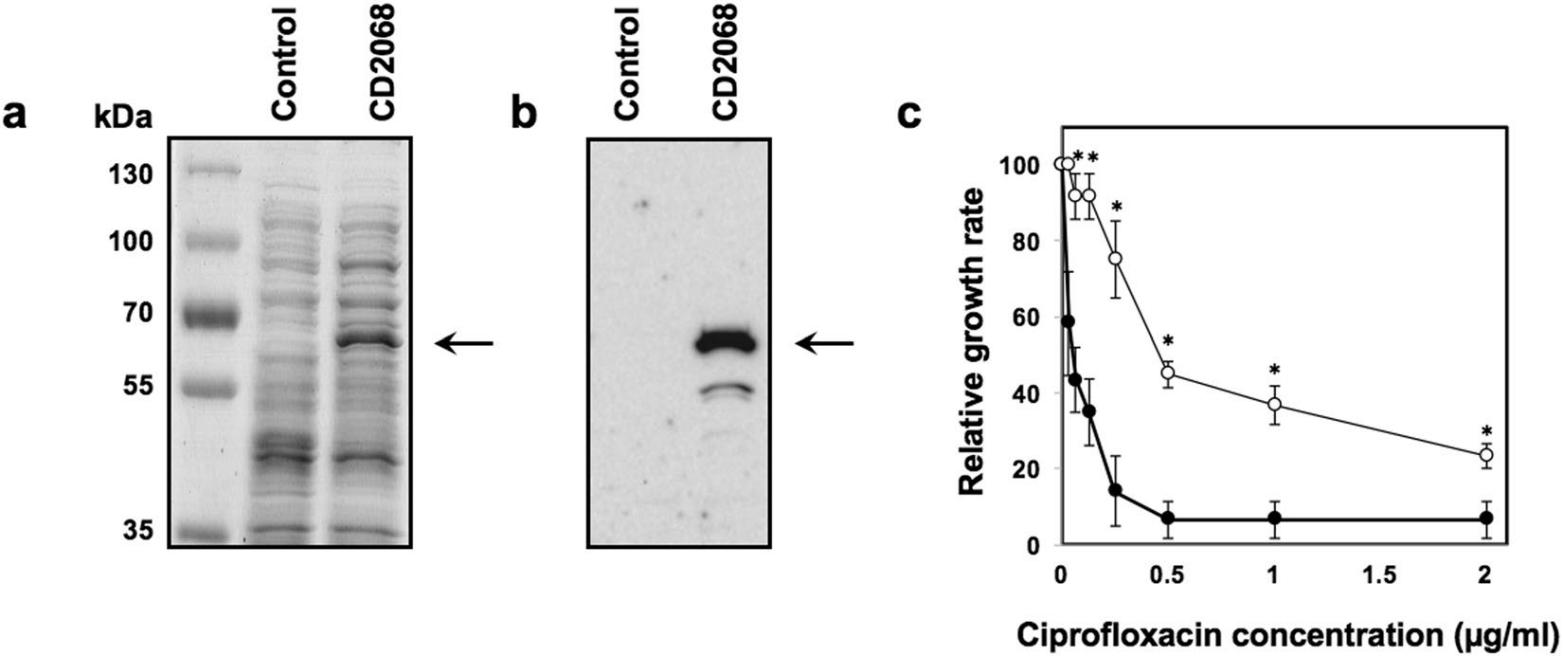
CD2068 conferred drug resistance in *E. coli*. (**a**) 10% SDS-PAGE stained with Coomassie Blue of total proteins prepared from an *E. coli* strain containing the pET28a empty plasmid (control), or overexpressing CD2068 (CD2068); 10 μg of total proteins were loaded into each lane. (**b**) Western blot of the histidine-tag CD2068 protein. 10 μg of total proteins were subjected to 10% SDS-PAGE and immunoblotting was performed against specific anti-His_6_ antibody. Arrows indicate the corresponding protein bands at ~60 kDa. (**c**) Relative growth rate of CD2068-expressing cells (○), and control cells (●) in the presence of ciprofloxacin at various concentrations ranging from 0 to 2 µg/ml. The OD600 values of the cultures were measured every 20 min for 6 h. The relative growth rate obtained in the absence of the drug was set to 100%. The error bars and the asterisks represent the standard errors and the statistical significance with the *p* values < 0.05, respectively.

To characterise the function of CD2068, the recombinant CD2068 containing a six-histidine tag at carboxyl terminus was heterologously expressed in *E. coli* at a level between 0.5 and 1% of total membrane protein, as determined by densitometric analysis of a Coomassie brilliant blue-stained SDS-PAGE gel. The size of the recombinant protein on 10% SDS-PAGE was determined to be approximately 60 kDa, which corresponds to the predicted molecular weight (Fig. 2a). The histidine-tagged CD2068 was further verified by immunoblotting using the specific anti-His_6_ antibody (Fig. 2b). This ~60 kDa band was absent in the control cells harboring the control pET28a plasmid. Consequently, the antibiotic susceptibility assays were performed, in which the growth of the cells in broth medium was followed over time. The results revealed a pronounced increment in the relative growth rate of the CD2068-expressing cells compared to the control cells in the presence of ciprofloxacin (Fig. 2c). The IC_50_ values were compared between the CD2068-expressing cells and the control cells in term of the relative resistance (Table 1). The maximal increase in the relative resistance (~10-fold) was observed for ciprofloxacin. Hence, the heterologous expression of the CD2068 protein conferred the resistance to all tested drugs, except gentamicin, in the *E. coli* cells.

**Table 1:** CD2068-associated drug resistance and drug-stimulated ATPase activity.

Antibiotics	IC_50_ (μg/ml)		Relative resistance	ATPase activity	
	Control	CD2068		Maximal stimulation (-fold)	SC_50_ (μM)
Ampicillin	8.50 ± 0.2	21.95 ± 0.7	2.6^*^	6.4	4.4 ± 0
Cefoxitin	0.06 ± 0.0	0.13 ± 0.1	2.2^*^	7.8	7.6 ± 1
Ciprofloxacin	0.05 ± 0.0	0.50 ± 0.1	10.0^*^	10.3	7.5 ± 0
Cloxacillin	1.15 ± 0.3	3.20 ± 0.4	2.8^*^	6.8	3.0 ± 0
Gentamicin	0.12 ± 0.0	0.13 ± 0.0	1.1	1.0	—
Levofloxacin	0.11 ± 0.1	0.50 ± 0.0	4.6^*^	7.9	4.5 ± 1
Roxithromycin	9.10 ± 0.8	18.10 ± 1.1	2.0^*^	3.7	2.9 ± 0
Vancomycin	29.60 ± 1.4	77.90 ± 1.7	2.6^*^	8.1	3.4 ± 1

SC_50_ values represent the concentrations of antibiotics at which the half-maximal stimulation of ATPase activity. Asterisks represent the *p* value of less than 0.05, which indicated that the mean IC_50_ values of CD2068-expressing *E. coli* cells are significantly different from that of the control cells.

### CD2068 mediated drug transport

**Figure 3:**
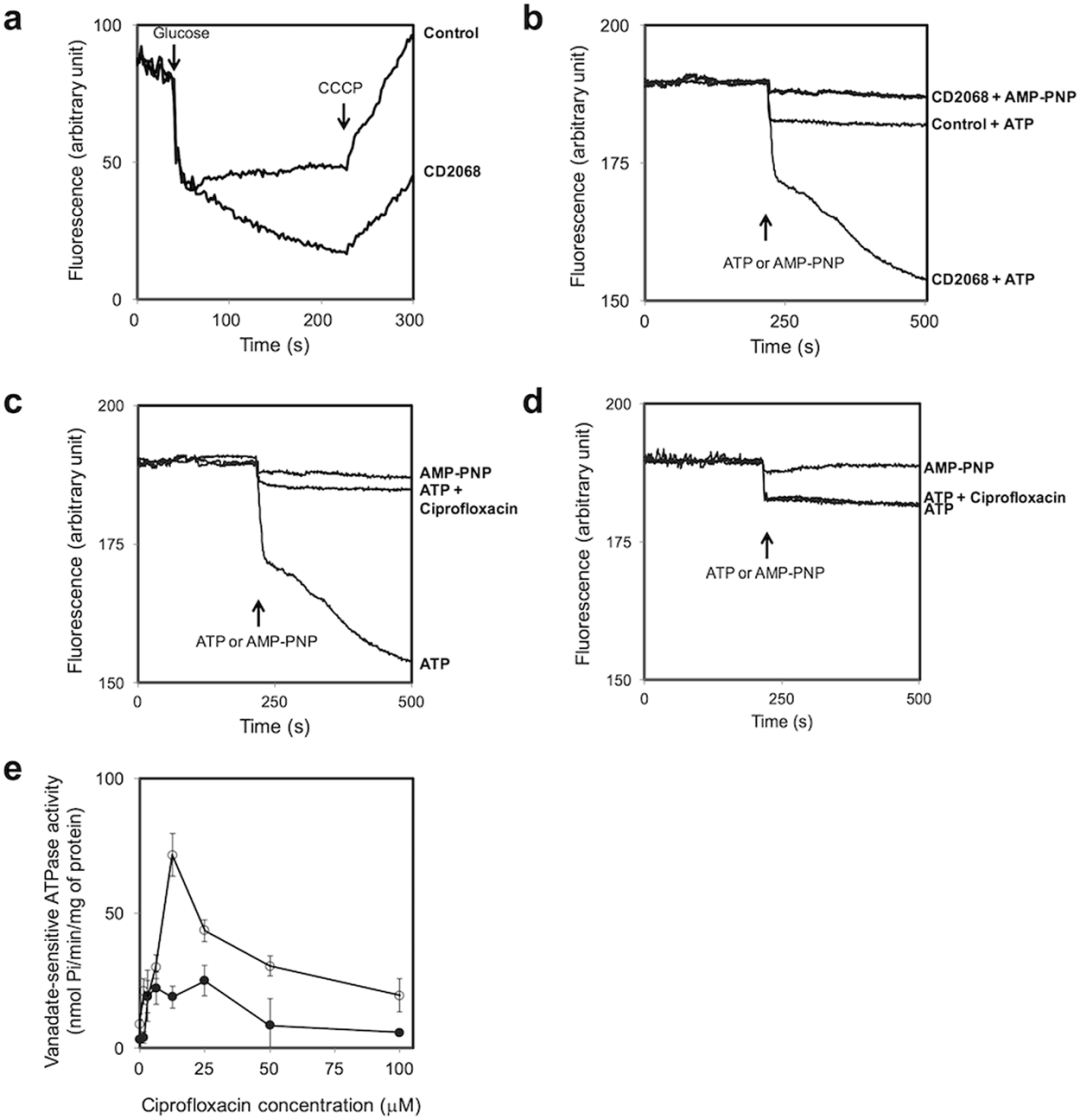
CD2068 mediated drug transport. (**a**) Ethidium transport in CD2068-expressing and control *E. coli* cells. The de-energized cells (OD_600_ = 0.5) of the CD2068-expressing and control cells were pre-equilibrated with 20 μM ethidium bromide, and 25 mM glucose was added (indicated by the first arrow) to initiate ethidium bromide efflux. CCCP at 10 μM was then added to the reaction, as indicated by the second arrow. (**b**) CD2068-mediated transport of Hoechst 33342 in inside-out membrane vesicles. After the steady state was reached, active transport of Hoechst 33342 was initiated by the addition of 5 mM ATP or nonhydrolysable ATP analogue AMP-PNP (indicated by the arrow). The IMV prepared from the *E. coli* cells with the empty plasmid were included as a control. (**c** and **d**) Competitive transport by ciprofloxacin. The ATP-dependent transport of Hoechst 33342 in IMV containing CD2068 (**c**) and control vesicles (**d**) was measured. Ciprofloxacin was added to the system at the final concentration of 5 μM prior to the addition of Hoechst 33342. (**e**) Ciprofloxacin stimulated the CD2068-associated ATPase activity in inside-out membrane vesicles. The vanadate-sensitive ATPase activity of inside-out membrane vesicles with CD2068 (○) and control membrane vesicles (●) in the presence of various concentrations of ciprofloxacin was measured as described in Methods. The error bars and the asterisks represent the standard errors and the statistical significance with the *p* values < 0.05, respectively.

To investigate the CD2068-mediated drug transport, we first assessed the transport of a typical substrate for ABC transporters, ethidium bromide in a real-time assay. Following passive diffusion into the bacterial cells, the ethidium exhibits increased fluorescence intensity upon binding to nucleic acids. The de-energised *E. coli* cells were pre-equilibrated in the ethidium bromide solution, and the direct ethidium efflux was measured following the addition of glucose. As shown in Fig. 3a, the energization of cells resulted in an increased rate of ethidium export for the CD2068-expressing cells compared to the control cells, which indicates that CD2068 displays an ATP-dependent transport activity. The efflux activity of CD2068 was further confirmed by determining the effect of the efflux pump inhibitor, carbonyl cyanide *m*-chlorophenylhydrazone (CCCP), on the accumulation of ethidium in the CD2068-expressing and control cells. Following the addition of CCCP, the level of ethidium bromide accumulation in the CD2068-expressing cells increased, suggesting that the CD2068-dependent ethidium efflux was blocked by CCCP. The increased ethidium accumulation following CCCP treatment was also observed in the control cells. It may be possible that CCCP could also inhibit the endogenous ABC transporters in the control cells.

The ability of the CD2068 protein to transport other fluorescent substrates for multidrug ABC transporters was also investigated using inside-out membrane vesicles, in which the nucleotide binding domains of CD2068 were flipped to the outside of the membrane surface. Hoechst 33342 is a membrane-permeable dye that is highly fluorescent in membrane environment but not in aqueous medium^26^. The addition of ATP resulted in a rapid quenching of Hoechst 33342 fluorescence in vesicles containing CD2068 while only slightly reduction was observed in the control vesicles (Fig. 3b). Moreover, no CD2068-mediated transport activity was observed when the non-hydrolysable ATP analogue, AMP-PNP, was added instead of ATP. To determine whether the CD2068 protein can recognize and transport antibiotics, the capacity of ciprofloxacin to compete with Hoechst 33342 transport was monitored in inside-out membrane vesicle. As shown in Fig. 3c, the presence of ciprofloxacin strongly inhibited the Hoechst 33342 transport activity by inside-out membrane vesicles containing CD2068. In contrast, no significant changes in the signal were observed in the control membrane vesicles in the presence of ATP alone or ATP with ciprofloxacin (Fig. 3d). These results indicate that the CD2068-mediated transport of Hoechst 33342 from the phospholipid bilayer into the aqueous lumen of the membrane vesicles is in an ATP dependent manner. Furthermore, the antibiotics including ciprofloxacin could be potential substrates for CD2068 as the effect of substrate competition between Hoechst 33342 and ciprofloxacin was observed.

The functionality of the expressed protein was also studied by measuring the ATPase activity in the inside-out membrane vesicles. As ATP hydrolysis is required for the ABC protein-mediated transport, the presence of potential substrates therefore should trigger the stimulation of the ATPase activity of the protein. The results revealed that, in contrast to the control vesicles, CD2068-containing membrane vesicles displayed an orthovanadate-sensitive ATPase activity that was stimulated up to ~10-fold in the presence of ciprofloxacin with a concentration required for half-maximal stimulation (SC_50_) of about 7.5 μM (Fig. 3e). The stimulation of ATPase activity was also observed in the presence of all tested antibiotics, except gentamicin, with different SC_50_ values (Table 1). These data strongly suggest that the drug-stimulated vanadate-sensitive ATPase activity is associated with CD2068 activity.

### *cd2068* gene disruption increased drug susceptibility in *C. difficile*

**Figure 4:**
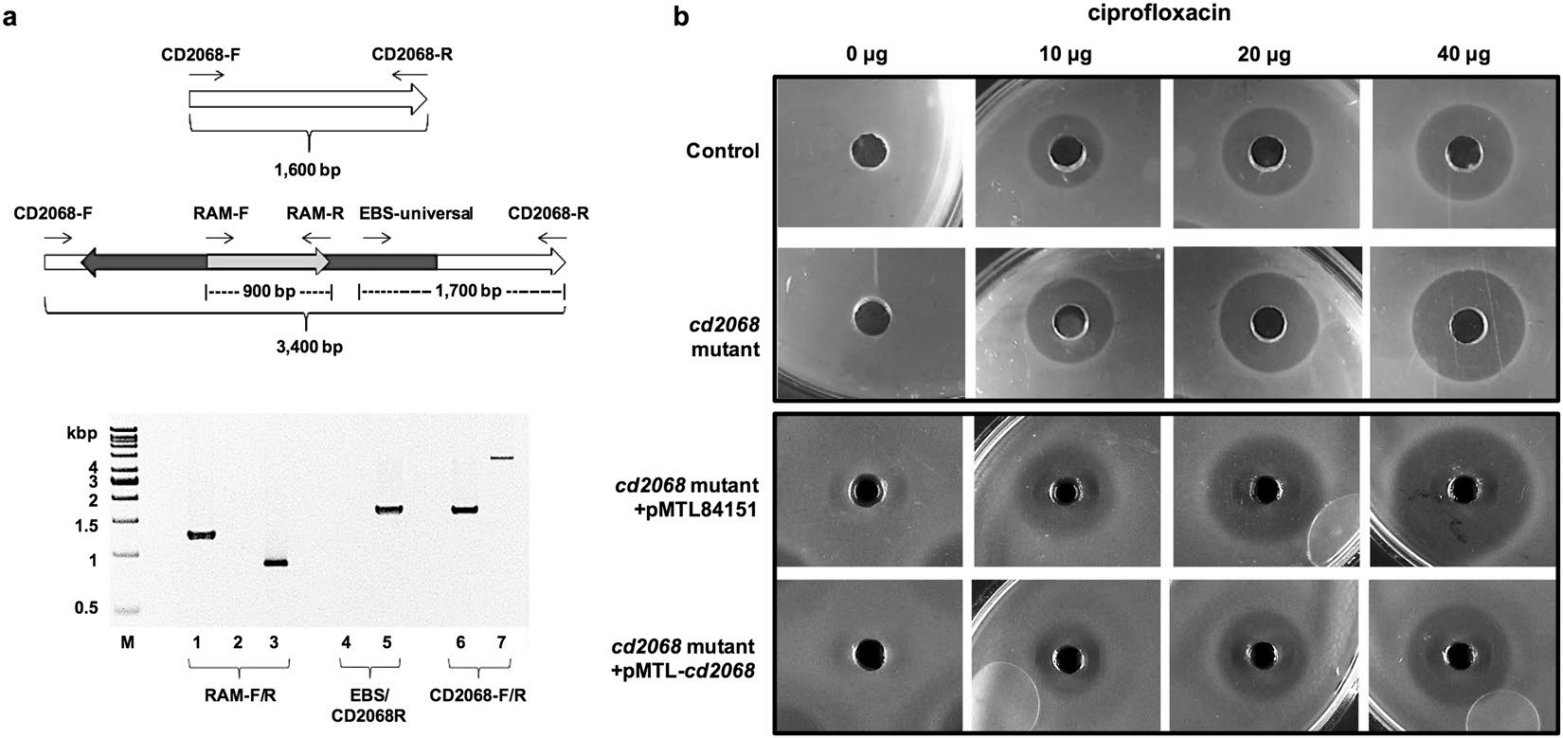
c*d2068 C. difficile* mutant exhibited increased drug susceptibility. (**a**) Verification of the *cd2068* mutant in *C. difficile*. (Top panel) schematic cartoons illustrated the *cd2068* gene before and after retro-transposition of the intron, arrows indicate primer used for verification. (Bottom panel) Agarose gel electrophoresis of PCR products amplified using corresponding set of primers. M represents DNA ladder; Lane 1 represents pMTL007C-E2:*cd2068* plasmid; Lanes 2, 4 and 6 represent *C. difficile* Δ*erm* (control); Lanes 3, 5 and 7 represent *cd2068* mutant. Lanes 1–3, 4–5, and 6–7 were amplified using RAM-F/RAM-R, EBS universal/CD2068-R, and CD2068-F/CD2068-R, respectively. (**b**) Disruption of *cd2068* in *C. difficile* enhanced antibiotic susceptibility, which was genetically complemented with the *cd2068* gene. Agar well diffusion assays were performed on the *cd2068* mutant versus control *C. difficile* as well as the *cd2068* mutants carrying pMTL84151 and pMTL-*cd2068* in the presence of various concentrations of ciprofloxacin.

In order to investigate the role of CD2068 on antibiotic resistance in *C. difficile*, the *cd2068* gene was disrupted using ClosTron gene knockout system^27^. In the *cd2068* mutant, the group II intron was inserted to the *cd2068* gene at the position 156 nucleotide in the coding sequence in the antisense orientation. The genotypes of the mutants were verified by PCR analysis (Fig. 4a). The RAM PCR was used to confirm the loss of group I intron that inactivated the *ermB*. The intron-exon junction PCR, which used gene specific and intron specific primers revealed the insertion of intron into the *cd2068* gene. The PCR products with the gene specific primers revealed the size of intron-inserted gene, which increased to ~3.4 kb compared to the ~1.6 kb of the wildtype. These PCRs confirmed that the group II intron was inserted into the *C. difficile* chromosome at the correct site and orientation, resulting in the inactivation of the *cd2068* genes. To examine the role of CD2068 on antibiotic resistance in *C. difficile*, the agar well diffusion and antibiotic susceptibility assays were performed. As shown in Fig. 4b, the *cd2068* mutant displayed higher sensitivity to ciprofloxacin compared to the control *C. difficile* cells. Furthermore, the *cd2068* gene complementation was introduced into the *cd2068* mutant to confirm that the observed drug sensitivity was caused by insertional inactivation of the *cd2068* gene. The results revealed that the *cd2068* mutant carrying pMTL-*cd2068* exhibited more resistance to ciprofloxacin compared to the *cd2068* mutant carrying the empty pMTL84151. Furthermore, the IC_50_ values of several structurally unrelated antibiotics were compared between the *cd2068* mutant and control *C. difficile* as well as the *cd2068* mutant carrying pMTL-cd2068 and pMTL84151 plasmids (Table 2). The relative sensitivity to the antibiotics was significantly increased up to 3-fold compared to the control strain, with an exception of vancomycin. The *cd2068* complemented strains also conferred higher resistance compared to the mutant control carrying the empty pMTL84151 plasmid. The roxithromycin was not included in this experiment as *C. difficile* 630 *Δerm* and *cd2068* mutant strains could not be grown in medium containing macrolide antibiotics.

**Table 2:** Antibiotic susceptibility profiles of *C. difficile* 630Δ*erm* control versus *cd2068* mutant and the *cd2068* mutants carrying pMTL84151 and pMTL-*cd2068*.

Antibiotics	IC_50_(μg/ml)		Relative sensitivity	IC_50_ (μg/ml)		Relative resistance
	Control	*cd2068* mutant		*cd2068* mutant + pMTL84151	*cd2068* mutant + pMTL-*cd2068*	
Ampicillin	29.84 ± 3.2	17.13 ± 0.7	1.7*	14.24 ± 0.6	25.56 ± 1.2	1.8*
Cefoxitin	33.69 ± 1.3	20.54 ± 2.7	1.6*	20.72 ± 1.1	46.03 ± 6.8	2.2*
Ciprofloxacin	12.26 ± 0.5	7.81 ± 0.2	1.6*	20.94 ± 0.6	34.50 ± 0.7	1.7*
Cloxacillin	16.90 ± 0.6	4.76 ± 0.3	3.6*	7.92 ± 0.3	9.73 ± 0.5	1.2*
Gentamicin	28.60 ± 2.3	22.72 ± 2.7	1.3*	107.83 ± 9.1	103.62 ± 15.3	1.0
Levofloxacin	7.28 ± 0.4	5.81 ± 0.7	1.3*	6.89 ± 0.8	10.03 ± 0.1	1.5*
Metronidazole	3.33 ± 0.5	2.37 ± 0.1	1.4*	2.36 ± 0.1	3.23 ± 0.1	1.4*
Vancomycin	2.01 ± 0.3	1.82 ± 0.2	1.1	3.38 ± 0.1	2.11 ± 0.1	0.6

Relative sensitivity refers to the fold difference in the IC_50_ values of the *cd2068* mutant compared to the control cells. Relative resistance refers to the fold difference in the IC_50_ values of the *cd2068* mutant carrying pMTL84151 compared to the *cd2068* mutant carrying pMTL-*cd2068*. Asterisks represent the *p* value of less than 0.05.

## Discussion

Antibiotic usage is known as a major risk factor for CDI. It has been proposed that antibiotics disrupt the intestinal microbiota, consequently leading to *C. difficile* colonization^7^. Metronidazole and vancomycin have been recommended as the standard treatment for CDI, however, the emergence of the resistance to these drugs results in the poor outcomes^8^. Alternative antibiotic therapies have been introduced to treat CDI, but the rise in resistance to these drugs following the treatment has been observed^28, 29^. Recently, we reviewed the mechanisms of resistance to antibiotics in *C. difficile* ^17^. While drug inactivation and target modification mediate the resistance to a specific class of antibiotics^13–15^, the multidrug resistance is associated with efflux mechanism^16, 17^. A study on an efflux pump belonging to the major facilitator superfamily in *C. difficile* revealed that this protein contributes to the resistance to ethidium bromide, safranin O, and erythromycin, when the *cme* gene was heterologously expressed in *Enterococcus faecalis* host cells^30^. Another sodium-dependent efflux pump belonging to the multidrug and toxic compound extrusion family, CdeA, has been linked to fluoroquinolone resistance in *E. coli* host cells^31^. Although the *cpr* operon, which includes the ABC transporter system, CprABC, has been associated with the resistance to antimicrobial peptides^24^, however, the detailed mechanisms of transport and the role of ABC transporters on multiple drug resistance in *C. difficile* still need to be fulfilled.

In this study, we aim to identify a novel drug efflux pump belonging to the ABC transporter superfamily. Among all the open reading frames present in the genome of *C. difficile* strain 630, a putative ABC transporter, CD2068, exhibited 63% and 68% amino acid sequence identity to fluoroquinolone resistance ABC transporter proteins CmpA in *C. hathewayi* ^22^ and CPE1506 in *C. perfringens* ^23^, respectively. Furthermore, the level of *cd2068* gene transcript was up-regulated following the exposure to antibiotics in the microarray analyses^25^. We therefore investigated the potential role of this putative ABC transporter involving in multiple antibiotic resistance in *C. difficile*. In the presence of various antibiotics, the gene expression of *cd2068* was enhanced as determined by the quantitative real-time PCR, consistent to the previous microarray transcriptomic data. These results support the notion that this ABC transporter could participate in drug resistance mechanism in *C. difficile*.

To study the function of this ABC efflux pump, we cloned and functionally characterized the CD2068 protein in *E. coli*. Firstly, CD2068 conferred the increased resistance to various structurally unrelated antibiotics when overexpressed in *E. coli*, with the exception for an aminoglycoside antibiotic gentamicin, indicating its ability to extrude these drugs out of the cells. Other ABC multidrug efflux pumps such as LmrA of *L. lactis* also have similar broad transport capacities^19^. Secondly, the energy-dependent transport of ethidium bromide was also observed in CD2068-expressing *E. coli* cells. The efflux of ethidium mediated by CD2068 was inhibited by the proton motive force inhibitor, CCCP, which drastically reduced the concentration of ATP by uncoupling protons across the membrane, pointing to the ATP-dependent transport activity of this protein. Thirdly, the active transport of Hoechst 33342 by CD2068 was demonstrated in inside-out membrane vesicles. In addition, the competition assays were also performed in the presence of ciprofloxacin. The competitive inhibition of CD2068-mediated transport of Hoechst 33342 by ciprofloxacin was demonstrated, suggesting the evidence of ciprofloxacin as a substrate competing with Hoechst 33342 for binding to common binding sites in CD2068. Finally, the ATPase activity of the CD2068 protein was shown to be stimulated by almost all tested antibiotics, with the highest elevation of ~10-fold in the presence of ciprofloxacin. The pattern of ATPase modulation in CD2068 is similar to that observed in other ABC proteins^26, 32^, in which there is a decline trend after the concentration of drugs that yields the maximal the ATPase activity. The explanations have been suggested that the high concentrations of drug may affect the binding to the protein or the membrane integrity, thereby providing less optimal conditions for CD2068 activity. Consistent to the antibiotic susceptibility results, gentamicin also did not stimulate the CD2068-associated ATPase activity, indicating that gentamicin may not be a transporting substrate of CD2068, hence the gentamycin resistance observed in *C. difficile* ^33^ might be mediated through other mechanisms. The increases in the CD2068-associated ATPase activity corresponded to the rise of relative resistance, further confirming that ATP-dependent CD2068 confers drug resistance in the recombinant strain.

To further validate the function of CD2068 in antibiotic resistance, the insertional inactivation and complementation of the *cd2068* gene were introduced into *C. difficile*. The agar well diffusion assay in the presence of ciprofloxacin showed the hypersensitivity to ciprofloxacin in the *cd2068* mutant, which could be genetically complemented, supporting the previous observations that this drug might be transported by CD2068. Despite the considerable effect of CD2068 on drug resistance and transport in *E. coli*, the susceptibility profiles of the *cd2068* mutant and the complemented strains revealed that CD2068 could significantly confer the resistance to certain antibiotics with a relatively low level in *C. difficile*. It might be explained by the facts that (i) CD2068 may be poorly expressed in its native host, *C. difficile*, under our experimental settings, however, it can still effectively pump drugs when overexpressed from a plasmid in *E. coli*; (ii) there are a total of 243 genes annotated to encode for putative ABC transporters present in the genome of *C. difficile* 630^34^ and they may exhibit an overlapping activity to compensate the loss of CD2086 in the mutant strain; and (iii) the resistance to certain antibiotics may also be mediated by other mechanisms in *C. difficile*.

In conclusion, we have identified CD2068 as the novel ABC drug efflux pump of *C. difficile* that is involved in resistance to multiple antibiotics. The protein can interact with various antibiotics and is able to transport them across the cell membrane. These findings suggest a novel molecular mechanism by which *C. difficile* can acquire a multidrug-resistant phenotype. The transportation through ABC transporters poses as one of mechanisms associated with multidrug resistance in *C. difficile* and could be a promising target for the development of novel antimicrobial therapeutic strategies.

## Methods

### Bacterial strains and culture conditions

*C. difficile* strain 630 was kindly gifted from Prof. Nigel Minton, University of Nottingham. *C. difficile* cells were anaerobically cultivated in Brain-Heart Infusion (BHI) medium at 37 °C. *E. coli* strains DH5α, BL21(DE3) pLysS and CA434 were cultured in Luria-Bertani (LB) broth at 37 °C. Chloramphenicol (25 μg/ml) and kanamycin (25 μg/ml) were added when necessary.

### Quantitative real-time polymerase chain reactions (qPCR)

*C. difficile* 630 was exposed to various types of antibiotics including ampicillin (1 μg/ml), cefoxitin (25 μg/ml), ciprofloxacin (1 μg/ml), cloxacillin (25 μg/ml), gentamicin (10 μg/ml), levofloxacin (1 μg/ml), roxithromycin (60 μg/ml), and vancomycin (0.25 μg/ml). Cells were harvested at the OD_600_ of 0.8 by centrifugation at 8,000 × *g* for 1 min. The cell pellets were suspended in RNAprotect bacteria reagent (Qiagen). The total RNA was extracted by sonication with TriZol reagent and reversely transcribed into complementary DNA using RevertAid Reverse Transcriptase (Thermo Scientific). The reverse transcription was carried out with a total volume of 20 μl containing 1 μg RNA sample, 100 pmol random primers, 4 μl 5× reaction buffer, 20 U RiboLock RNase Inhibitor (Thermo Scientific), 2 μl 10 mM dNTP, and 200 U RevertAid Reverse Transcriptase. The mixture was incubated at 42 °C for 16 h followed by heat-terminated reaction at 70 °C for 10 min. The qPCR assays were carried out using a qPCR SYBR green PCR master mix, FastStart Universal SYBR Green Master (Rox) from Roche. Cycling conditions were 10 min at 94 °C, 40 cycles of 30 s at 94 °C, 1 min at 57 °C, and 30 s at 72 °C. The primers for amplification of the *cd2068* gene include the forward primer: 5′-G GAC GAA CCT ACA AAC CAC-3′, and the reverse primer: 5′-GCC AAT TGA CTT GAC TCA TAC C-3′. The *rpsJ* 30 S ribosomal housekeeping gene was used as an internal control^35^. Relative gene expression was calculated with the standard errors from 4 independently conducted experiments and *t*-test was used for statistical significance testing between relative gene expression of antibiotics in treated and control cells.

### Cloning of *cd2068* in *E. coli*

The coding region of *cd2068* (CD630_20680: Accession YP_001088582.1) was amplified using the forward primer (CD2068-F) 5′-CAT GCC ATG GGC ATG TTA CAA GTT ACA G-3′to introduce an *Nco*I site (underlined) at the 5′ end and the reverse primer (CD2068-R) 5′-CGG AAT TCG GTT TAT CTT TTC CAT ACA TT-3′to introduce an *Eco*RI site (underlined) at the 3′ end of the gene. The fragment was digested with *Eco*RI and *Nco*I and the product was purified before ligation into the pET28a expression vector at the corresponding sites, allowing the addition of six consecutive histidine residues on the carboxyl terminus of the protein to yield pCD2068. The obtained plasmid was then initially transformed into *E. coli* DH5α and subsequently into BL21(DE3) pLysS. DNA sequencing was performed to ensure that only the intended changes had been introduced.

### Protein expression and inside-out membrane vesicle preparation

Overproduction of the recombinant protein was performed in auto-induction medium as previously reported^36^. Briefly, a single colony of *E. coli* BL21(DE3) pLysS harboring the pCD2068 plasmid was inoculated into sterile ZYM-5052 medium at 25 °C for 16 h. Subsequently, 1% of the total culture was transferred into new ZYP-5052 and kanamycin and chloramphenicol (25 μg/ml) were supplied when appropriate. The culture was incubated at 25 °C until its optical density at 600 nm reached 1.0 and then maintained the culture at 16 °C for the next 14 h. Bacterial lysis and inside-out membrane vesicle preparation were conducted as previously described^26^. Approximately 10 μg of cell lysate protein was subjected to 10% SDS-PAGE and immunoblotting using rabbit anti-his-tag antibody (1:500), followed by goat anti-rabbit HRP conjugated antibody (1:1000) and enhanced chemiluminescence for signal development.

### Construction of *cd2068 C. difficile* mutant and complementation

*cd2068* was disrupted in the erythromycin sensitive host strain *C. difficile* 630Δ*erm* using the ClosTron gene knock-out system^27^. The group II intron for *cd2068* was designed and inserted into pMTL007C-E2 vector by DNA2.0. The disruption vector was then transferred to *C. difficile* 630Δ*erm* via conjugation using *E. coli* CA434 donor strain as previously described^27^. Briefly, 1 ml of overnight culture of *E. coli* CA434 was harvested at 4,000 × *g* for 2 min and mixed with 200 μl of *C. difficile* 630Δ*erm*. A total of 20 μl of cell mixture was dropped onto BHI plate, following by incubation at 37 °C for 24 h under anaerobic condition. Bacterial mixtures were harvested by flooding 1 ml of Tryptone Yeast extract (TY) broth, and appropriate dilution of cell suspension was spread onto the *C. difficile* selective medium, Cycloserine Cefoxitin Frutose agar (CCFA), supplemented with 15 μg/ml of thiamphenicol. Erythromycin (2.5 μg/ml) was then used to select *C. difficile* 630Δ*erm* integrants. The insertion in the *cd2068* gene was verified by PCR and DNA sequencing. For the complementation, the *cd2068* coding sequence together with the 5′ upstream promoter region (~400 bp) was amplified by PCR from the genomic DNA of *C. difficile* 630 using the primers Com_2068 F (5′-CCG CTC GAG TTT GTA TGA ACT GTG ATG TAT GTA CTT ATC CTG C-3′) with an introduction of *Xho*I restriction site (underlined), and Com_2068 R (5′-AAG GAA AAA AGC GGC CGC TTA atg atg atg atg atg atg TTT ATC TTT TCC ATA CAA TTT TAG CTA ATT G-3′) with modified *Not*I site (underlined) and a histidine_6_ tag (lowercase letter). The amplified product was then verified by sequencing and cloned into the modular vector pMTL84151 to generate pMTL-*cd2068*, which was then conjugated into *C. difficile cd2068* mutant. The presence of the *cd2068* gene in the complement strain harboring pMTL-*cd2068* was verified by PCR.

### Antibiotic susceptibility assays

To compare the initial growth rate, the CD2068-expressing and the control *E. coli* cells were cultivated in LB medium supplemented with various concentrations of antibiotics including ampicillin, cefoxitin, ciprofloxacin, cloxacillin, gentamicin, levofloxacin, roxithromycin, and vancomycin. The OD_600_ values of the cultures were measured every 20 min for 6 h in a plate reader. The concentrations that inhibited the maximum growth rate by 50% (IC_50_) were calculated by Probit analysis. For *C. difficile*, the *cd2068* mutant, the Δ*erm* control, and the *cd2068* mutants harboring the empty pMTL84151 and pMTL-*cd2068* were subjected to similar experiments in anaerobic environments. Agar well diffusion assays were also performed on *C. difficile* to verify the findings.

### Ethidium bromide transport in intact cells

Overnight culture of *E. coli* was harvested and washed three times with ice-cold 50 mM KPi (pH7.0) containing 5 mM MgSO_4_. To deprive cell metabolic activity, the cell suspensions were incubated for 30 min at 30 °C in the presence of 0.5 mM dinitrophenol and washed three times with the same buffer. The cell pellets were then resuspended to an OD_600_ of 0.5 in 2 ml of KPi buffer. After 1 min of incubation, 2 μM ethidium bromide was added, allowing the detection of facilitated ethidium influx into the cell for 1 min. The efflux process was initiated when 20 mM glucose was added into the solution and maintained for 3 min. The inhibitor of oxidative phosphorylation, CCCP (10 μM) was added to stop the reaction. Fluorescence was measured in a Jasco FP-6200 spectrofluorometer at the excitation and emission wavelengths of 500 and 580 nm, respectively with slit widths of 5 and 10 nm.

### Hoechst 33342 transport in membrane vesicles

Inside-out membrane vesicles (0.5 mg of total membrane) were diluted in 2 ml of ice-cold 100 mM KPi buffer (pH7.0) containing 5 mM MgSO_4_, 0.1 mg/ml creatine kinase, and 5 mM phosphocreatine. After incubation at 30 °C for 1 min, Hoechst 33342 prepared in KPi buffer was added to a final concentration of 0.25 μM, and the binding of the dye to the membrane vesicles was followed by spectrofluorometry at the excitation and emission wavelengths of 355 and 457 nm, respectively, with slit widths of 2.5 nm (Jasco FP-6200) until a steady state was reached. Subsequently, ATP (or AMP-PNP, a non-hydrolysable ATP analogue for the control experiments) dissolved in KPi buffer was added to a final concentration of 2 mM, and the fluorescence intensity was again followed until a new steady state was reached.

### ATPase assay

Inside-out membrane vesicles prepared from the CD2068-expressing and control cells were diluted to ice-cold 1 mg/ml in the 20 mM HEPES (pH7.4) containing 5 mM MgSO_4_, and 5 mM ATP. ATPase assays were conducted at 30 °C for 10 min in a 96-well plate with various concentrations of drugs in a reaction volume of 10 μl/well. The ATPase reactions were terminated by addition of 40 μl of a mixture containing 0.048% (w/w) ammonium heptamolybdate tetrahydrate, 6.6% (v/v) concentrated sulfuric acid, 0.01% (w/w) potassium antimonyl tartrate, and 0.42% (w/w) ascorbic acid. Subsequently, 150 μl of water was added and incubated at 30 °C for 30 min in the dark. The released phosphate was determined colorimetrically at 690 nm in a microplate reader. ATPase activity measured in the presence of 1 mM orthovanadate were obtained in parallel and subtracted from the background prior to calculation.

### Data availability statement

No datasets were generated or analysed during the current study.
